# Assessment of Spinal Range of Motion and Musculoskeletal Discomfort in Forklift Drivers. A Cross-Sectional Study

**DOI:** 10.3390/ijerph18062947

**Published:** 2021-03-13

**Authors:** Juan Rabal-Pelay, Cristina Cimarras-Otal, Noel Marcen-Cinca, Andrés Alcázar-Crevillén, Carmen Laguna-Miranda, Ana Vanessa Bataller-Cervero

**Affiliations:** 1 Faculty of Health Science, Universidad San Jorge, Autovía A-23 Zaragoza-Huesca Km. 299, 50830 Villanueva de Gállego, Spain; jrabal@usj.es (J.R.-P.); nmarcen@usj.es (N.M.-C.); avbataller@usj.es (A.V.B.-C.); 2 Hospital MAZ, Avda. Academia General Militar, 74, 50015 Zaragoza, Spain; aalcazar@maz.es; 3 BSH Electrodomésticos España S.A., Pol. Industrial Otallana, Avenida La Industria, 49, 50016 Zaragoza, Spain; Carmen.Laguna@BSHG.com

**Keywords:** office workers, lumbar ROM, spine, forklift operators, low back discomfort

## Abstract

Forklifts are commonly used in industrial supply chains to transport heavy loads. Forklift drivers have the risk of developing musculoskeletal discomfort derived from the movement pattern required at work. This research aimed to investigate the spinal range of motion (ROM) and musculoskeletal discomfort of forklift drivers and compare it with a control group. Forklift drivers (39 males) and office workers (31 males) were recruited to assess cervical, thoracic, and lumbar ROM with an electronic double inclinometer. Additionally, musculoskeletal discomfort was registered with the Cornell Discomfort Musculoskeletal Questionnaire. Forklift drivers showed a higher cervical discomfort and ROM of lateral lumbar bending than office workers. Both groups reported lower ROM in cervical and lumbar lateral bending on the right side versus the left side. No differences of asymmetry were reported for any variable between groups. Specific exercise programs may correct these mobility imbalances.

## 1. Introduction

Forklifts are commonly used in industrial supply chains, where transport of heavy goods and loads lifting are involved. Those vehicles are responsible for traumatic accidents and musculoskeletal disorders (MSDs) for the forklift driver [1,2,3].

Some of the risk factors of MSDs for those individuals are repetitive trunk twisting, sideways trunk bending and rotation, exposure to whole-body vibrations [1,3,4,5,6], neck flexion and rotation, especially during reverse driving [3,7], neck extension without support, and static sitting positions [8].

The most common body regions affected by MSDs in forklift drivers are the lumbar spine [2,3,8,9,10], cervical spine [7,8,9,11], shoulders, and forearms [8,9]. Other studies defend the necessity of high-quality epidemiologic research about forklift drivers’ injuries [10].

Throughout the workday, forklift drivers repeatedly rotate their necks and also bend to the side to get a better view when loading and unloading their vehicles [7]. Babapour et al. explained that when forklift drivers are handling pallets of supplies, the work is performed on the right side of the driver’s cabin at different heights [12]. This requires the operator to support twisted trunk postures with a rotated neck and shoulder complex. These postures are overused to overcome the visibility restrictions of the driver’s cabin [12].

It may be possible that the repeated movements performed by forklift drivers, which are conditioned by the features of the forklift truck (seat, steering wheel, controls, rearview mirror, etc.), could cause spinal range of motion (ROM) adaptations, ROM decrease in the non-dominant side, and decompensations between the left and right side. Kramárová et al. suggested that decreased ROM and musculoskeletal discomfort are symptoms of the beginning of MSDs for forklift operators [8]. Past studies observed how repeated and specific movements could generate adaptations in a joint’s ROM [13,14,15,16].

The present study aims to assess the cervical, thoracic, and lumbar ROM and spinal musculoskeletal discomfort of forklift drivers and compare the results to a control group of office workers in order to investigate if there are adaptations caused by the forklift driving. Office workers share a prolonged sitting workday with forklift drivers, but they do not need to twist or bend the spine constantly or suffer whole-body vibrations. Previous research conducted with workers from different manufacturing industries has studied low back pain, comparing blue-collar and white-collar workers [17,18,19,20,21].

## 2. Materials and Methods

### 2.1. Study Design

This was an observational cross-sectional study. Every procedure was conducted in accordance with the principles of the World Medical Association’s Declaration of Helsinki. Each participant was informed about the nature of the study, the voluntariness of the participation, the aims of the project, as well as its possible adverse effects, and signed informed consent. Participants could abandon the study at any time. The protocol was approved by the committee of ethics in research of the regional government (C.I. PI17/090).

Forklift drivers were recruited in a home appliances manufacturing company. Office workers were recruited from the informatics and management section of an associated hospital.

The medical service of the manufacturing company recruited the volunteer workers that met the inclusion criteria, and office workers were recruited through informative mail sent to all the workers. The forklift model was EFG 216K (Jungheinrich, Hamburg, Germany). Participants spent most of their eight-hour workday in prolonged sitting and had a minimum of 5 years of experience in the job position. They were asymptomatic adults without diagnosed back pathologies in the previous 3 months. The exclusion criteria were scoliosis diagnosed by a doctor, surgeries, or structural alterations of the spine and/or severe pathologies that prevented them from performing their functions in the workplace.

After signing the informed consent, the clinical information about the subjects was collected: age, experience in the job, height, weight, left- or right-handedness, as well as diagnosed pathologies related to the spine that could be considered exclusion criteria. Then, the subjects were assessed in the biomechanics laboratory of the associated hospital by the same researcher.

### 2.2. Outcome Measures and Measure Method

#### 2.2.1. Musculoskeletal Discomfort

The musculoskeletal discomfort perception in the last week was assessed utilizing the Cornell Musculoskeletal Discomfort Questionnaire (CMDQ), Spanish adaptation and validation [22]. The scoring guidelines of the CMDQ appear on the official website [23]. The total score was calculated by multiplying the above “frequency score” (0, 1.5, 3.5, 5, 10) by the “discomfort score” (1,2,3) and the “work interference score” (1,2,3). The musculoskeletal discomfort perception is an important subjective dimension to be evaluated in MSDs in workers. Previous studies have evaluated the musculoskeletal discomfort in workers with the CMDQ [24,25,26,27].

#### 2.2.2. Range of Motion

The degrees of motion in cervical flexion (CF), cervical extension (CE), left/right cervical lateral bending (CLB), left/right cervical rotation (CR), thoracic flexion (TF), left/right thoracic rotation (TR), lumbar flexion (LF), lumbar extension (LE), and left/right lumbar lateral bending (LLB) were measured by an electronic system of double inclinometry Ned MCV/IBV (IBV, Spain), following the protocol defined by the system. The device offers a 0.1° resolution and uncertainty of 1.7°. The device is factory calibrated and offers a measurement range of flexion-extension of −40° to 100°, lateral flexion of −70° to 70°, and rotation of −100° to 100°. The software employed in the assessment was the NedDiscapacidad/IBV version 3.0 (IBV, Valencia, Spain).

**Figure 1:**
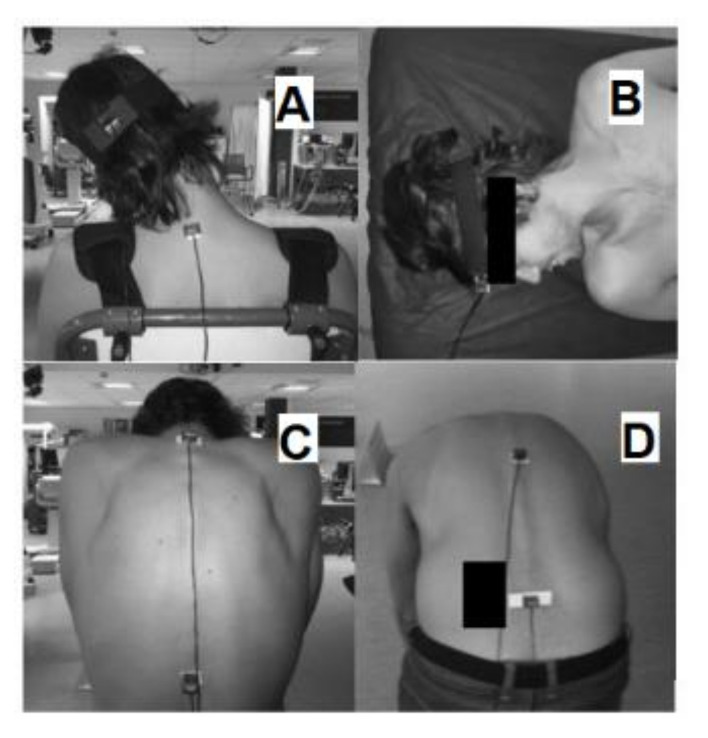
Range of motion measures. (**A**): Cervical lateral bending (CLB); (**B**): Cervical rotation (CR); (**C**): Thoracic flexion (TR); (**D**): Lumbar flexion (LF).

The skin of the subject was cleaned with alcohol before placing the sensors. Double-sided adhesive tape was used to fix the sensors to the skin. For the cervical ROM assessment, the subject was sitting. One sensor was located in the vertex of the head (highest point in the sagittal plane of the head), fastened with an elastic strap. The second one was fixed to the spinous process T1 for the CF, CE, and CLB measurements (Figure 1A). The CR was measured with the subject lying in a supine position, with sensors fixed to the forehead (Figure 1B). The TF and TR were evaluated with the subject in a standing position. The sensors were placed in spinous processes T1 and T12. The TR was measured from a dorsal flexion with the arms in front of the chest (Figure 1C). The sensors were located in the spinous process T12 and the center of the sacrum for LF, LE, and LLB (Figure 1D). The order of the evaluation was cervical, thoracic, and lumbar ROMs. Subjects performed each movement 3 times. The maximum ROM was recorded.

Asymmetry variable was calculated comparing right and left movements in CR, CLB, TR, and LLB ROMs.

### 2.3. Statistical Analysis

To categorize the results, the variables were described in number and percentage or mean and standard deviation (SD) if they were qualitative or quantitative respectively. The normality of variables was analyzed by applying the Shapiro–Wilk test.

An independent *t*-test was used to compare the baseline variables of the groups studied.

Inter-group analysis was performed with an independent samples *t*-test. A two-factor (job and motion) ANOVA test with a paired sample in the second factor was used to analyze the difference between the right and left in the same subjects in lateral bending and rotation movements in cervical, thoracic, and lumbar spine in both groups. An ANOVA test was performed to observe the differences in asymmetry in variables with right-left sides between the work groups. The confidence interval was adjusted by Bonferroni.

A Spearman’s correlation test was used to relate the variables of musculoskeletal discomfort, baseline characteristics, and ROM values in each group. A Pearson correlation test was performed to relate baseline characteristics and ROM values in both groups.

The level of signification was set to 0.05. All statistical procedures were completed on IBM™ SPSS™ Statistics (version 21, IBM Corporation, Somers, New York, NY, USA).

## 3. Results

In total, 70 workers participated in the study, 39 forklift drivers and 31 office workers. All participants were males. The age range in the forklift drivers’ group was 28 to 58 years, while in the office workers group, it was 31 to 58 years. Characteristics of the participants are shown in Table 1. No inter-group differences were observed in age, weight, height, or body mass index (BMI). There was a significant difference in the number of years worked, which was higher in office workers.

### 3.1. Inter-Group Analysis: Forklift Drivers versus Office Workers

**Figure 2:**
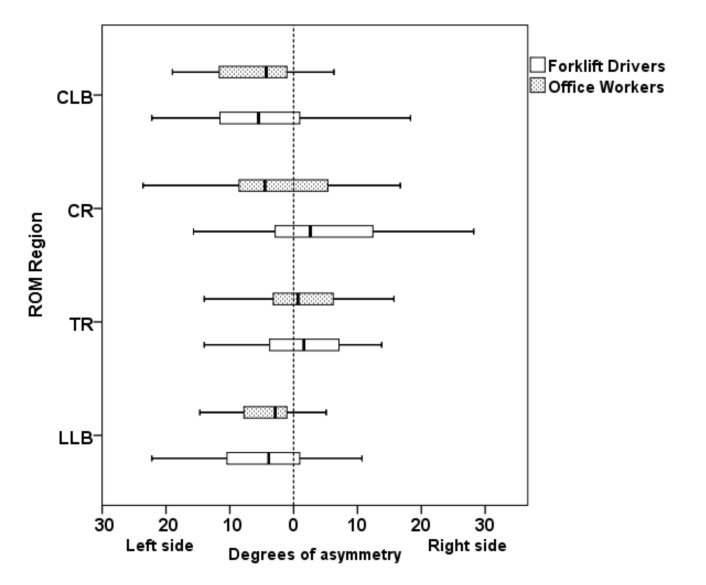
Degrees of asymmetry (right side range of motion (ROM)–left side ROM, between groups in different body regions. Asymmetry = right side ROM–left side ROM. CLB: Cervical lateral bending. CR: Cervical rotation. TR: Thoracic rotation. LLB: Lumbar lateral bending.

Forklift drivers showed a greater ROM in right LLB (*p* < 0.01) and left LLB (*p* < 0.01) than office workers. Moreover, forklift drivers had a higher discomfort score in the cervical spine (*p* < 0.05) than office workers (Table 2). The cervical CMDQ variable had non-normal distribution in both groups. The ANOVA test showed that the level of asymmetry (right side ROM–left side ROM) was not different between the work groups for any of the variables studied (Figure 2).

### 3.2. Intra-Group Analysis: Left versus Right Movements

The intra-group analysis showed a significantly smaller ROM in right CLB in relation to the left CLB in both groups, forklift drivers (*p* < 0.01) and office workers (*p* < 0.01). Additionally, forklift drivers (*p* < 0.01) and office workers (*p* < 0.01) obtained a smaller ROM in right LLB in comparison to the left LLB.

### 3.3. Correlation Analysis

A positive correlation was found between age and years worked (r = 0.555, *p* = 0.000) in the total sample. Age, years of service, or BMI did not correlate with any discomfort variable in both groups.

In the forklift drivers’ group, the age showed a negative correlation with CF (r = −0.368, *p* = 0.021), left CLB (r = −0.477, *p* = 0.002), left CR (r = −0.357, *p* = 0.026), and left LLB (r = −0.433, *p* = 0.006). The CF variable had non-normal distribution in this group.

In the office workers’ group, age showed a negative correlation with left CLB (r = −0.400, *p* = 0.026), left CR (r = −0.421, *p* = 0.018), TF (r = −0.369, *p* = 0.041), right TR (r = −0.389; *p* = 0.033), and left TR (r = −0.480, *p* = 0.007). Right and left CLB variables did not have a normal distribution in this group.

Years of service in the company showed a negative correlation with CF (r = −0.403, *p* = 0.013) in the forklift drivers’ group; meanwhile, in the office workers, the years of service in the company did not correlate with any variables. CF variable had non-normal distribution in the forklift drivers’ group.

In the forklift drivers’ group, CMDQ score did not correlate with any variables, whereas in the office workers’ group, cervical CMDQ showed a negative correlation with CE (r = −0.476, *p* = 0.007), and spine CMDQ also showed a negative correlation with CE (r = −0.438, *p* = 0.014). Cervical CMDQ and spine CMDQ did not have a normal distribution in the office workers’ group.

## 4. Discussion

ROM results showed that forklift drivers had greater LE and bilateral LLB than office workers. It may be possible that the repetitive movements needed to drive the forklift truck caused these differences in the spinal ROM between groups. Previous studies described ROM adaptations in other joints due to repetitive movements, as shown in the shoulder rotation of tennis, baseball, and softball players [13,14,15,16].

In relation to the intra-group differences between left and right ROM, the analysis showed a greater left CLB and LLB compared to the right side in both groups, forklift drivers and office workers. The level of asymmetry is similar between groups. On the other hand, the absolute right and left LLB values were significantly higher in the forklift drivers’ group. The results showed an increase in spinal ROM of forklift drivers, which could be related to the labor demand movement, but without increasing the degree of asymmetry between the left and right side concerning the control group. Gombatto et al. found greater asymmetry in LLB mobility in adults with low back pain than those without it [28,29]. With a larger sample, it would be interesting to categorize the ROM results depending on the suffering of spinal discomfort or not.

The asymmetry comparison showed a non-significant difference (*p* = 0.052) in cervical rotation between work groups. The authors expected that forklift drivers showed a larger CR on the right side because of the driving needs. Babapour et al. [12] studied forklift drivers’ postures based on video observations during different driving tasks. Babapour et al. [12] concluded that in handling tasks with pallets on high shelves, forklift drivers leaned forward, the torso was rotated to the right side, and the head was rotated to the right and tilted to the left side [12].

Rislund et al. [30] quantified the physical workload on the neck, shoulders, and upper extremities of forklift drivers as an effect of steering systems. Rislund et al. [30] found large differences in most of the results between the right and left sides, the left side being more active [30]. The results of the present study did not show significant differences in thoracic rotation between forklift drivers and office workers. In line with this result, no differences in ROM and muscle activity were observed between forklift drivers and office workers in trunk rotation in a sitting posture [6].

The significant differences between the left and right in CLB and LLB in office workers could be due to the different muscle activation, because of the position of the computer screen or documents during task work, as suggested by previous studies [31]. Some risk factors for developing musculoskeletal disorders in office workers, such as positioning and height of the screen [32], keyboard position [33], or crossing legs while in a sitting position [34], could be a possible factor affecting ROM. There is also evidence of a reduction in lumbar mobility in subjects older than 40 years old [25].

Forklift drivers showed greater cervical discomfort compared to office workers. This could be generated by the higher demand for spinal movement during the workday, as well as the whole-body vibration of the forklift. These results are consistent with the study of Flodin et al. [7], who concluded that being a forklift operator was associated with an increased risk of neck pain when compared with office workers. Holding the head in an unnatural position resulted in significantly increased risks for neck pain [7].

The spinal and cervical CMDQ scores correlated negatively with CE ROM in office workers. This may be due to the subjects with more discomfort refusing to move in the direction of painful movements. These results differ partially from those suggested by Ariëns et al. [11], who pointed out that sitting at work for more than 95% of working time seems to be a risk factor for neck pain, and there is a trend for a positive relation between the degrees of neck flexion and neck pain [11]. Workers of both job groups presented reduced CE ROM when compared with other research in worker populations [35,36].

Despite the fact that the differences between forklift drivers and office workers concerning musculoskeletal lumbar discomfort have not been significant, and that there is no correlation between being a forklift driver and lumbar CMDQ score, previous studies indicated that there is a causal relationship between forklift operation and lumbar pain [2,3,9]. Lumbar pain is most prevalent in forklift drivers and driving postures in which the trunk is rotated or flexed forward [2,9].

In a study conducted in Japan with forklift drivers, it was observed that the initial prevalence of low back pain was 63%, significantly higher than that observed in other manual workers (32%) and that of office workers (22%) [17]. In the present study, forklift drivers and office workers presented a prevalence of low back discomfort of 50% and 36% respectively. In total, 65% of forklift drivers reported some discomfort in the spine area, while this percentage was 60% for office workers.

Similarly to forklift drivers, office workers spend most of the workday in a sitting position. They also suffer from MSDs, such as non-specific neck pain [33,37,38,39], hand and wrist symptoms [38], and lumbar pain [32,37,40,41]. Waongenngarm et al. pointed out that the prolonged postural loading of the spine while sitting can reduce joint lubrication and fluid content of intervertebral discs and increase stiffness, which can be detrimental to back health [42]. An incorrect position of the computer screen was found to be an important risk factor for neck and lumbar pain in office workers [32]. Other authors described different risk factors for neck pain in those individuals, such as inaccurate keyboard position, low work task variation, self-perceived medium/high muscular tension [33], or neck rotation and self-reported neck extension [43].

It would be interesting to assess the discomfort and ROM in retired workers, to check if there are differences when workers stop their professional activity and do not need to continue with the repetitive spinal movements. In the control group of office workers, one limitation is the non-register of the keyboard position and the frequency of crossing legs while in a sitting position during their workday. These issues should be taken into consideration in future studies. Due to the sample only comprising male workers, results cannot be transferred to the female population.

It would also be important to study the possible causes of differences found in ROM, by analyzing the work tasks in a real work environment in both groups with movement sensors and/or video recording. It could even be possible to perform diagnostic tests to assess the structural changes in the spine in a prospective way.

## 5. Conclusions

Lumbar lateral bending ROM and cervical musculoskeletal discomfort values were higher for the forklift drivers when compared to office workers. There is also a greater left cervical lateral bending and left lumbar lateral bending compared to the right side in both groups. The analysis performed could help to design a program of compensatory exercises to prevent muscular imbalances and musculoskeletal alterations for workers in the future. Prescription of preventive exercise and education on the use of the rear-view mirror or installation of a camera in the forklift cabin could be interesting to avoid overusing the mobility of the spine in forklift drivers while driving backwards.

## Figures and Tables

**Table 1:** Baseline variables.

Characteristics	Total N = 70	Forklifts Drivers N = 39	Office Workers N = 31
Age (years), Mean (SD)	41.9 (7.7)	40.9 (8.3)	43.1 (6.9)
Handedness, n (%)			
Right	66 (94.3)	35 (88.6)	31 (100)
Left	4 (5.7)	4 (11.4)	0 (0)
Years of service in the company, Mean (SD)	14.3 (8.0)	12.2 (8.6) ^a^	17.0 (6.4) ^a^
Height (m), Mean (SD)	1.75 (0.06)	1.74 (0.06)	1.76 (0.06)
Weight (kg), Mean (SD)	78.6 (12.8)	78.5 (13.0)	78.8 (12.8)
Body Mass Index, Mean (SD)	25.5 (3.6)	25.7 (3.4)	25.2 (3.8)

^a^: *p* ≤ 0.05 inter-group comparison.

**Table 2:** Mean and standard deviation (SD) of the range of motion (°) and Cornell Musculoskeletal Discomfort Questionnaire (CMDQ) scores in forklift drivers and office workers.

	Forklifts Drivers N = 39	Office Workers N = 31	Asymmetry Comparison
Range of Motion	Mean ± SD (^o^)	Mean ± SD (^o^)	*p*
Cervical flexion (CF)	39.2 ± 12.5	39.6 ± 11.4	-
Cervical extension (CE)	36.6 ± 13.6	38.2 ± 14.0	-
Right cervical lateral bending (Right CLB)	37.3 ± 11.8 ^b^	32.4 ± 8.7 ^b^	0.608
Left cervical lateral bending (Left CLB)	42.4 ± 7.5 ^b^	38.6 ± 9.3 ^b^	
Right cervical rotation (Right CR)	76.4 ± 16.9	73.0 ± 12.6	0.052
Left cervical rotation (Left CR)	73.4 ± 10.1	75.8 ± 11.4	
Thoracic flexion (TF)	33.4 ± 12.7	32.2 ± 8.3	-
Right thoracic rotation (Right TR)	34.2 ± 7.9	31.5 ± 8.1	0.715
Left thoracic rotation (Left TR)	33.1 ± 9.3	29.6 ± 7.6	
Lumbar flexion (LF)	52.4 ± 10.5	50.9 ± 11.9	-
Lumbar extension (LE)	36.4 ± 13.6	32.6 ± 16.6	-
Right lumbar lateral bending (Right LLB)	37.3 ± 11.8 ^a,b^	28.2 ± 10.5 ^a,b^	0.772
Left lumbar lateral bending (Left LLB)	41.8 ± 7.6 ^a,b^	33.3 ± 12.1 ^a,b^	
**Musculoskeletal Discomfort**	Mean ± SD	Mean ± SD	-
Cervical CMDQ	10.5 ± 18.1 ^a^	1.1 ± 1.8 ^a^	-
Thoracic CMDQ	8.3 ± 22.2	2.5 ± 7.8	-
Lumbar CMDQ	13.9 ± 27.1	6.7 ± 15.7	-
Spine CMDQ	32.8 ± 52.3	10.3 ± 18.2	-

^a^: *p* ≤ 0.050 inter-group comparison, ^b^: *p* ≤ 0.050 intra-group comparison between right and left sides. CMDQ: Cornell Musculoskeletal Discomfort Questionnaire score.

## Data Availability

The data are not publicly available due to privacy policy on participant data.
